# Microbial manipulation in atopic dermatitis

**DOI:** 10.1002/ctm2.828

**Published:** 2022-04-22

**Authors:** Portia Gough, Muhammad B. Khalid, Stella Hartono, Ian A. Myles

**Affiliations:** ^1^ National Institute of Allergy and Infectious Disease National Institutes of Health Bethesda Maryland USA

**Keywords:** atopic dermatitis, ATx201, Roseomonas, *Staphylococcus*

1

*Staphylococcus aureus* is a Gram‐positive bacteria found on the skin of approximately 20–30% of healthy subjects, but on 30–100% of patients with atopic dermatitis (AD).^1^ Recent reviews^2^, ^3^ have eloquently detailed a myriad of *S. aureus‐*secreted toxins, enzymes, and cell‐surface‐associated antigens which contribute to AD pathogenesis (Figure 1). In brief, proteins, such as clumping factor B and fibronectin binding proteins, promote the adhesion of *S. aureus* to the stratum corneum. *Staphylococcal* Protein A can activate proinflammatory nuclear factor kappa B (NF‐κB) signaling through direct engagement of tumor necrosis factor receptor 1 (TNFR1).^2^, ^3^ Lipoprotein and lipoteichoic acid induce TSLP in human keratinocytes via Toll‐like receptors (TLR‐) 2 and 6,^2^ and phenol‐soluble modulins also induce proinflammatory cytokines in human keratinocytes.^2^, ^3^ Additionally, secreted δ‐toxin promotes mast cell degranulation via phosphoinositide 3‐kinase (PI3K) and Ca^2+^ influx‐dependent mechanisms.^4^ *Staphylococcus aureus* enterotoxins and toxic shock syndrome toxin can influence disease in many ways such as: acting as superantigens, promoting clonal T‐expansion and inflammatory cytokine release; inducing IgE isotype switching in B‐cells; directly activating mast cells and basophils; or stimulating secretion of itch‐inducing interleukin 31 (IL‐31).^2^, ^3^ *Staphylococcal* α‐toxin not only promotes NLRP3 activation and IL‐31 production but additionally compromises the keratinocyte layer by altering E‐cadherin integrity.^2^, ^3^, ^4^ Although no single virulence factor correlates with AD prevalence or severity, AD exacerbations correlate with differences in the specific combinations of virulence factors within distinct lineages of *S. aureus* (called clonal complexes).^3^ Recent reviews^2^, ^3^ have eloquently detailed a myriad of *S. aureus‐*secreted toxins, enzymes, and cell‐surface‐associated antigens which contribute to AD pathogenesis (Figure 1). In brief, proteins, such as clumping factor B and fibronectin binding proteins, promote the adhesion of *S. aureus* to the stratum corneum. *Staphylococcal* Protein A can activate proinflammatory nuclear factor kappa B (NF‐κB) signaling through direct engagement of tumor necrosis factor receptor 1 (TNFR1).^2^, ^3^ Lipoprotein and lipoteichoic acid induce TSLP in human keratinocytes via Toll‐like receptors (TLR‐) 2 and 6,^2^ and phenol‐soluble modulins also induce proinflammatory cytokines in human keratinocytes.^2^, ^3^ Additionally, secreted δ‐toxin promotes mast cell degranulation via phosphoinositide 3‐kinase (PI3K) and Ca^2+^ influx‐dependent mechanisms.^4^ *Staphylococcus aureus* enterotoxins and toxic shock syndrome toxin can influence disease in many ways such as: acting as superantigens, promoting clonal T‐expansion and inflammatory cytokine release; inducing IgE isotype switching in B‐cells; directly activating mast cells and basophils; or stimulating secretion of itch‐inducing interleukin 31 (IL‐31).^2^, ^3^ *Staphylococcal* α‐toxin not only promotes NLRP3 activation and IL‐31 production but additionally compromises the keratinocyte layer by altering E‐cadherin integrity.^2^, ^3^, ^4^ Although no single virulence factor correlates with AD prevalence or severity, AD exacerbations correlate with differences in the specific combinations of virulence factors within distinct lineages of *S. aureus* (called clonal complexes).^3^

**FIGURE 1:**
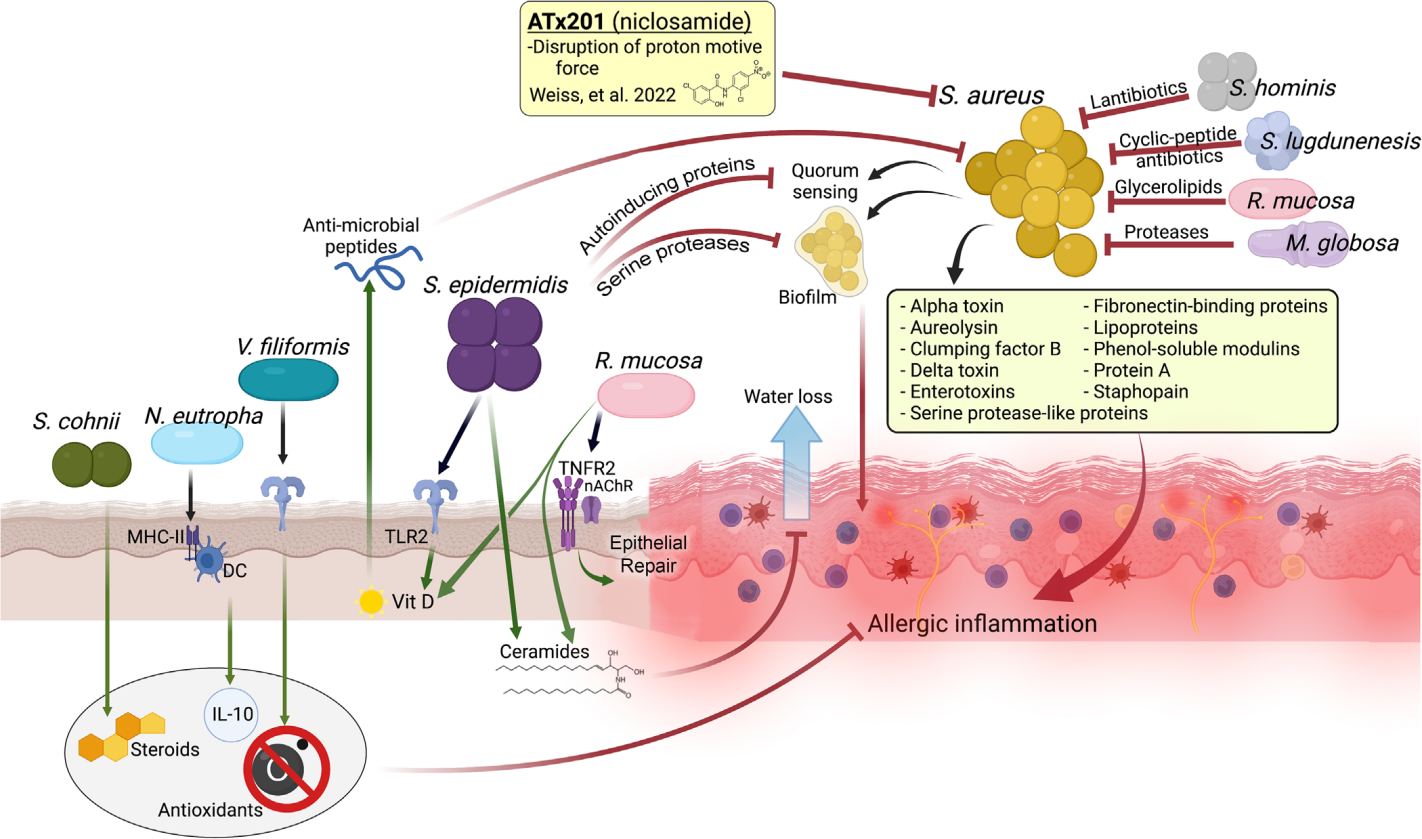
Summary of microbial influence on allergic skin disease. Overview of influence of health‐associated commensals on skin homeostasis and *S. aureus* control along with influence of *S. aureus* on allergy inflammation. MHC‐II, Major Histocompatibility Complex 2; TLR2, Toll‐like receptor 2; TNFR2, tumor necrosis factor receptor 2; nAChR, nicotinic acetylcholine receptor; IL‐10, interleukin‐10; Vit D, vitamin D. Image generated by Ian Myles using BioRender.com

While the deleterious impacts of *S. aureus* are well established, AD is definitively non‐communicable. The realization that a non‐contagious disease like AD could not be caused by a highly contagious organism like *S. aureus* fostered the appreciation for the protective role of other microbes in skin homeostasis (Figure 1). The best studied is *Staphylococcus epidermidis*, which was first postulated to support host production of vitamin D through basal activation of TLR2.^5^ Additionally, *S. epidermidis* induces host ceramides through the production of sphingomyelinase^6^ and directly inhibits *S. aureus* growth and colonization through induction of host antimicrobial peptides like cathelicidin; production of biofilm‐inhibiting serine proteases; and production of autoinducing peptides which disrupt quorum sensing.^7^ Other coagulase‐negative *staphylococci* (CoNS) species that influence skin health include *Staphylococcus cohnii*, which improves outcomes in mouse models of skin disease through alteration of host steroid pathways.^8^ *Staphylococcus lugdunensis* inhibits *S. aureus* growth through the production of cyclic peptide antibiotics while select isolates of *Staphylococcus hominis* reduce the growth of *S. aureus* through the production of lantibiotics.^7^

*Roseomonas mucosa*, a Gram‐negative commensal isolated from healthy skin, also induces vitamin D and cathelicidin^9^; additionally, *R. mucosa* produces glycerophopholipids that inhibit *S. aureus* and induce host epithelial repair through enhancing the cholinergic potentiation of TNFR2 signaling.^10^ *Vitreoscilla filiformis*, found in thermal springs, has been reported to reduce skin inflammation through a combination of antioxidant induction and TLR2‐mediated induction of host defensins.^7^ Ammonia oxidizing bacteria like *Nitrosomonas eutropha* inhibits allergy‐associated inflammation through upregulation of IL‐10, potentially through inhibition of Major Histocompatibility Complex type II (MHC‐II) expression on dendritic cells.^11^ One fungal commensal, *Malassezia globosa*, also inhibits *S. aureus* growth through specific proteases.^7^

Despite the expanding arsenal of potential anti‐*S. aureus* options, the utility of topical antimicrobial therapies targeting *S. aureus* remains questionable. A meta‐analysis of bleach baths did not support early hopes that the practice could control *S. aureus* and/or improve AD symptoms.^3^ Use of topical antimicrobials applies selective pressure favoring the type of resistance identified by studies demonstrating higher rates of mupirocin‐ and fusidic acid‐resistant *S. aureus* in children with AD compared to healthy children.^12^, ^13^ The genetic basis of fusidic acid resistance was most frequently due to either chromosomal mutations or plasmid‐derived *qac* genes which could also confer resistance to many clinical antiseptics.^12^ Thus, treatments that target *S. aureus* have not been shown to yield long‐term decolonization. Yet even in the absence of resistance, antibiotics have not been shown to improve the symptoms of AD, and treatment guidelines explicitly recommend against the use of topical or oral antibiotics unless clinical signs of frank cellulitis are present.^3^, ^14^ Furthermore, attempting to curtail *S. aureus* through topical use of CoNS‐containing probiotics^3^ presents an experimental risk of infecting patients with the treatment strain.^15^

However, Weiss et al.,^16^ aim to succeed where prior anti‐*S. aureus* strategies have failed through several unique innovations. In their study, the authors demonstrated the potent anti‐*staphylococcal* activity of niclosamide, a traditional antihelminthic drug. In vitro, niclosamide demonstrated a narrow minimal inhibitory concentration (MIC; 0.125–0.5 μg/mL) against *S. aureus* strains with dose‐dependent kinetics, showing bacteriostatic behavior at lower concentrations which progressed to bactericidal activity at higher concentrations. In contrast to comparators, niclosamide led to immediate bacterial growth arrest along with pH‐dependent inhibition of upstream biosynthesis and protonophore activity. These observations suggest a novel antibacterial mechanism through proton carrier activity, which reduces cytoplasmic pH and dissipates proton motive force. This unique mechanism may inform the authors’ findings that, unlike other antimicrobials tested (including mupirocin and fusidic acid), niclosamide did not induce detectable spontaneous resistance mutations and was less likely to induce resistance in both serial passage and murine models using methicillin‐resistant strains.

The authors then present findings from their randomized, double blinded, phase 2 trial of adults with mild‐severe AD treated once or twice daily for 7 days with topical 2% niclosamide on one half of their body and placebo on the other. Overall, treatment was well tolerated though unrelated gastrointestinal adverse effects were seen in 9% of participants. By day 7, active treatment led to significant reduction in *S. aureus* culture yield (twice‐daily versus placebo: 94.8 versus 38.9%; once daily versus placebo: 50 to 33.3%). Importantly, the *S. aureus* MIC for niclosamide remained unchanged and the skin Shannon diversity index increased during the study. Together, these suggest that niclosamide led to selective *staphylococcal* killing without the emergence of resistance or adverse impacts on other commensal microbiota. While treatment resulted in only modest improvement of clinical disease scores, a longer duration of treatment will be needed to properly assess clinical utility. Therefore, Weiss et al. may have found a new purpose for niclosamide as an anti‐*S. aureus* agent. Their identified novel mechanism, superior resistance escape, and favorable selectivity of impact on the microbiota all offer hope that niclosamide may soon serve a role in AD treatment.

## CONFLICT OF INTEREST

The authors have no conflict of interest to declare.
